# Human monoclonal IgG selection of *Plasmodium falciparum* for the expression of placental malaria-specific variant surface antigens

**DOI:** 10.1111/j.1365-3024.2009.01097.x

**Published:** 2009-06

**Authors:** J SOERLI, L BARFOD, T LAVSTSEN, N L BERNASCONI, A LANZAVECCHIA, L HVIID

**Affiliations:** 1 Centre for Medical Parasitology, Department of International Health, Immunology and Microbiology, University of Copenhagen DenmarkDenmark; 2 Department of Infectious Diseases, Copenhagen University Hospital (Rigshospitalet), University of CopenhagenDenmark; 3 Institute for Research in BiomedicineBellinzona, Switzerland

**Keywords:** *human monoclonal IgG*, *PfEMP1*, Plasmodium falciparum, *pregnancy*, *VAR2CSA*

## Abstract

*Pregnancy-associated*Plasmodium falciparum *malaria (PAM) is a major cause of morbidity and mortality in African women and their offspring. PAM is characterized by accumulation of infected erythrocytes (IEs) that adhere to chondroitin sulphate A (CSA) in the placental intervillous space. We show here that human monoclonal IgG antibodies with specificity for variant surface antigens (VSA) specifically expressed by CSA-adhering IEs (VSA_PAM_) can be used* in vitro *to select parasites from nonpregnant donors to express VSA_PAM_ and that this selection for VSA_PAM_ expression results in preferential transcription of var2csa. The results corroborate current efforts to develop PAM-specific vaccines based on VAR2CSA.*

## INTRODUCTION

Children living in areas of stable *Plasmodium falciparum* transmission acquire substantial protective immunity against malaria during the first decade of life. Protection is mediated to a large extent by variant surface antigen (VSA)-specific IgG. Nevertheless, women in such areas remain highly susceptible to *P. falciparum* infection if they become pregnant, as the parasites can switch to expression of particular VSA (called VSA_PAM_), which allow infected erythrocyte (IE) sequestration in the placenta, but which are not compatible with parasite survival in a nonpregnant host. Acquired immunity to PAM is mediated by VSA_PAM_-specific IgG that either opsonizes IEs for phagocytosis or interferes with chondroitin sulphate A (CSA)-specific adhesion of IEs (1). VAR2CSA, which is a member of the *P. falciparum* erythrocyte membrane protein 1 (PfEMP1) family of VSA, appears to be the dominant or only pregnancy-associated *Plasmodium falciparum* malaria (PAM) type VSA in the *P. falciparum* genome, and is therefore the main target of current efforts to develop a vaccine against PAM (2). To assist this work, we have developed a panel of VSA_PAM_-specific human IgG1 monoclonal antibodies (3), which can opsonize VSA_PAM_-expressing IEs for phagocytosis and interfere with IE adhesion to CSA (Barfod *et al.* unpublished data). These antibodies can be used to enrich for VSA_PAM_ expression in parasites not expressing VSA_PAM_ but obtained from women with PAM (3). In the present study we used two of the above-mentioned monoclonal antibodies to select for VSA_PAM_ expression in parasites from nonpregnant donors. One antibody (PAM1·4) was chosen because it appears to react with most or all parasites expressing VSA_PAM_. The other antibody (PAM8·1) was chosen because it reacts with a well-defined, but inter-clonally variant, epitope in the DBL3X domain of VAR2CSA.

## MATERIALS AND METHODS

### Monoclonal antibodies

We used eight VSA_PAM_-specific monoclonal IgG1 antibodies generated as described elsewhere (3; 4). VSA_PAM_ is defined here as IE surface-expressed VSA, which are significantly better recognized by plasma IgG from *P. falciparum*-exposed multigravidae than from sympatric men, and where the recognition by plasma IgG from these men is not significantly different from recognition by plasma IgG from nonexposed controls. In contrast, typical non-PAM VSAs are better recognized by plasma IgG from *P. falciparum*-exposed adults than from nonexposed donors, without marked sex-dependent differences. Seven of the monoclonal antibodies used here are specific for inter-clonally variant epitopes in either the DBL3-X or the DBL5-ɛ domain of VAR2CSA (3). The exact specificity of the last antibody (PAM1·4) remains undefined, but it appears to recognize a conformational, and possibly discontinuous, epitope in VAR2CSA that is difficult to reproduce in recombinant constructs (3). PAM1·4 recognizes VSA_PAM_ expressed by most or all *P. falciparum* genotypes, whereas the VAR2CSA DBL3-X epitope recognized by PAM8·1 is present in some, but not all *P. falciparum* clones (3). Both PAM1·4 and PAM8·1 can opsonize VSA_PAM_-expressing IEs for phagocytosis and interfere with their adhesion to CSA (Barfod *et al.* in preparation).

### Malaria parasites

We used the two long-term *in vitro*-adapted parasites 3D7 and HB3. The 3D7 clone was originally derived from NF54 parasites isolated from a Dutch girl near Amsterdam airport (5). It was chosen here because it can be selected for expression of VSA_PAM_ that react with PAM1·4 but not PAM8·1 (3). HB3 was cloned from the Honduras I/CDC parasite strain (6), and was chosen because it can be selected for expression of VSA_PAM_ reactive with PAM1·4 and PAM8·1. All parasites were grown in 0^+^ erythrocytes as described (3).

### Antibody selection for VSA_PAM_ expression

Selection for VSA_PAM_ expression was done essentially as described elsewhere (7). In brief, monoclonal PAM1·4 or PAM8·1 antibodies were immobilized on Protein A-coated magnetic beads (Dynal) and mixed with 3D7- or HB3-IEs. IEs adhering to the antibody-coated beads were isolated in a strong magnetic field and subsequently returned to *in vitro* culture. Selection protocol was repeated when multiplication of the antibody-selected parasites allowed it. VSA expression was assessed by flow cytometry analysis as described elsewhere (8). We used plasma from 10 *P. falciparum*-exposed multigravidae, 10 sympatric men, and 10 nonexposed controls to assess the sex-specificity of VSA recognition. According to the original criteria (9), sex-specific recognition requires that (i) levels of IE-surface reactive IgG are significantly higher in *P. falciparum*-exposed multigravidae than in sympatric men, and that (ii) the difference in IgG levels in *P. falciparum*-exposed men and nonexposed controls is not statistically significant. We used term plasma from 30 *P. falciparum*-exposed women (10 pregnant for the first time, 10 for the third time, and 10 for the fifth time) to assess parity-dependency, which requires a statistically significant relationship between IgG levels and number of pregnancies in *P. falciparum*-exposed women (9). Parasite isolates were only considered to express VSA_PAM_ if plasma IgG recognition of IEs was both sex-specific and parity-dependent.

### Analysis of *var* gene transcription

Late-stage IEs were isolated by magnetic separation as described (8) and returned to culture overnight to obtain ring-stage IEs. Genomic DNA was isolated with a QIAamp blood kit (Qiagen), and total RNA extracted (TRIzol, Invitrogen) and treated with DNase I (Invitrogen) for 30 min (10). The absence of DNA in RNA samples was confirmed as described (11). Reverse transcription was performed using Superscript II (Invitrogen) and random hexamer primers, followed by real-time PCR to quantify *var* transcript abundances as described (11). We used primer pairs specific for the 59 *var*genes in the 3D7 genome (12) and for 45 *var* genes in the HB3 genome (Table S1 in Supporting Information). The latter were tested on genomic DNA dilutions to ascertain appropriate fragment size, melting temperature, and amplification efficiency compared to the internal control *seryl-tRNA synthetase*. All primer pairs varied less than two Ct values from that of the internal control and amplified single fragments of the expected sizes.

### Statistical analysis

Differences in antibody levels among plasma from *P. falciparum*-exposed multigravidae, *P. falciparum*-exposed men, and nonexposed control donors were analysed by one-way anova, or by one-way analysis of ranks for non-normal distributed data. If statistically significant (*P* < 0·05) overall differences were detected, significant pair-wise differences were isolated by Holm-Sidak's or Dunn's test for normal- and non-normal-distributed data, respectively. Parity-dependency was analysed by Pearson product moment correlation.

## RESULTS

### Human monoclonal IgG can select *Plasmodium falciparum*-IEs for expression of VSA_PAM_

**Figure 1:**
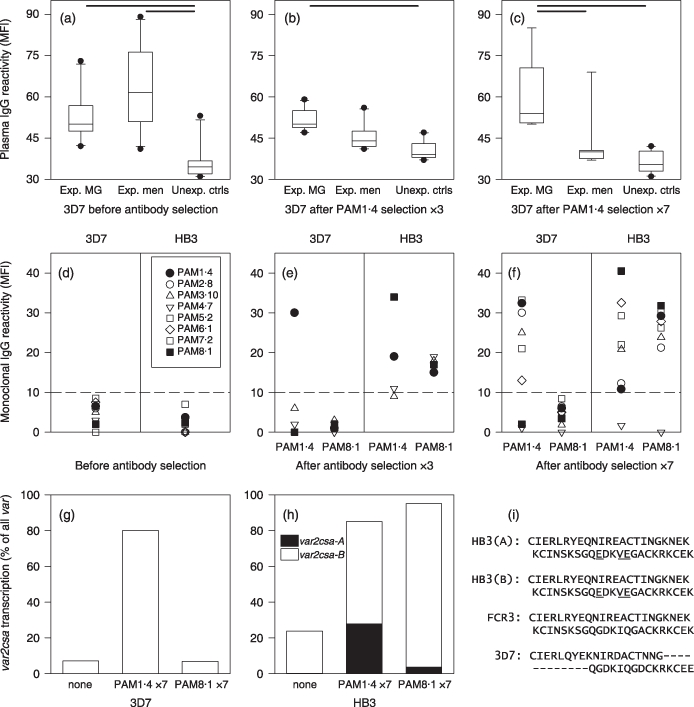
*Changes in VSA expression and*var *gene transcription following selection for expression of VSA_PAM_ reactive with human monoclonal antibodies.*Recognition of IEs by plasma IgG from 10 *P. falciparum*-exposed multigravidae (Exp. MG), 10 sympatric men (Ex. men) and 10 nonexposed controls (Unexp. ctrls) before selection (a), and after three (b) or seven (c) rounds of selection by human monoclonal IgG antibody PAM1·4. Results in panels A-C are presented as medians (horizontal line), central 50% of data points (boxes), central 80% of data points (whiskers) and outliers (•). In addition, statistically significant (*P* < 0·05) pair-wise differences are indicated by heavy horizontal bars along the top of the panels. Recognition of IEs by human monoclonal VSA_PAM_-specific IgG antibodies before selection (d), and after three (e) or seven (f) rounds of selection. Results in panels D–F are presented as individual data points. Negative cut-off, defined as the upper level of recognition of unselected parasites, is indicated as a dashed horizontal line. The proportion of *var2csa* transcripts among all *var* transcripts in 3D7 (g) and HB3 (h) before selection and after seven rounds of selection. Panel (i) shows the amino acid sequence of the PAM8·1-specific region of VAR2CSA DBL3-X in the two VAR2CSA paralogs in HB3, FCR3, and 3D7 parasites. Amino acid differences between the two HB3 sequences and the PAM8·1-reactive FCR3 sequence are underlined.

The VSA expressed on the surface of unselected 3D7- and HB3-IEs were significantly better recognized by plasma IgG from *P. falciparum*-exposed donors of both sexes than by IgG from nonexposed donors, whereas levels in exposed men and multigravidae were not significantly different from each other. Furthermore, IE surface-reactive IgG levels did not depend on parity (Table 1 and Figure 1a). Thus, unselected 3D7 and HB3 both showed typical non-PAM-type plasma antibody recognition patterns of IE surface-expressed VSA. This was confirmed by the nonreactivity of 3D7- and HB3-IEs with all eight VSA_PAM_-specific monoclonal antibodies (Figure 1d). When VSA expression was re-assessed after three rounds of selection (about 6 weeks after the first round of selection), the recognition of 3D7- and HB3-IEs selected on either PAM1·4 or PAM8·1 all showed an indeterminate VSA phenotype, where one but not both criteria for sex-specific antibody recognition were met (Table 1 and Figure 1b). PAM8·1-selected 3D7 remained nonreactive with the four monoclonal antibodies used for testing at this time (PAM1·4, PAM3·10, PAM4·7, and PAM8·1), whereas the other three parasite lines showed reactivity with at least one of them (Figure 1e). These results suggested that further rounds of selection might lead to definite VSA_PAM_ expression, at least for PAM1·4-selected 3D7, PAM1·4- and PAM8·1-selected HB3. Indeed, PAM1·4-selected 3D7, as well as PAM1·4- and PAM8·1-selected HB3 had all acquired a typical sex-specific and parity dependent VSA_PAM_ expression pattern after four additional rounds of selection (Table 1 and Figure 1c), and reacted with all the monoclonal antibodies except the VAR2CSA DBL5-ɛ-specific PAM4·7 (Figure 1f). In contrast, 3D7 selected seven times for reactivity with PAM8·1 retained an indeterminate sex-specificity pattern also seen after three rounds of selection, did not acquire the parity-dependent pattern typical of VSA_PAM_-expressing lines (Table 1), and did not react with any of the eight VSA_PAM_-specific monoclonal antibodies (Figure 1f). Thus, PAM8·1 could not be used to select 3D7 for VSA_PAM_ reactivity, consistent with the absence of the predicted epitope for this antibody in the VAR2CSA DBL3-X domain of this parasite (3).

**Table 1:** Changes in plasma antibody recognition pattern of infected erythrocytes following selection with VSA_PAM_-reactive human monoclonal antibodies

		Rounds of selection for antibody recognition					
Parasite	Antibody^a^	0		3		7	
		Sex-specificity^b^	Parity-dependency	Sex-specificity	Parity-dependency	Sex-specificity	Parity-dependency
3D7	PAM1·4	No	No: *P*(*r*) = 0·87	Indeterminate	n.d.	Yes	Yes: *P*(*r*) = 0·002
	PAM8·1			Indeterminate	n.d.	Indeterminate	No: *P*(*r*) = 0·41
HB3	PAM1·4	No	No: *P*(*r*) = 0·55	Indeterminate	n.d.	Yes	Yes: *P*(*r*) = 0·006
	PAM8·1			Indeterminate	n.d.	Yes	Yes: *P*(*r*) = 0·037

a Human monoclonal VSA_PAM_-specific antibody used for selection.

b Determination of sex-specificity involves a two-step procedure: first establishment that IE surface-reactive IgG levels in at least one of the three donor categories (exposed multigravidae, exposed men, and unexposed controls) differs from at least one other category. Only if this is the case (as it was here: *P* < 0·001 in all cases), can sex-specificity be confidently assessed by *post hoc* testing for significant (*P* < 0·05) pair-wise differences. The result of this *post hoc* testing is indicated here as No: none of the two *post hoc* criteria for sex-specificity were met, Indeterminate: only one of the criteria was met. Yes: both criteria were met. See Materials and Methods for further details.

### Acquisition of VSA_PAM_ expression following antibody-selection is associated with selective transcription of *var2csa*

Transcripts of *var2csa* (PFL0030c) constituted 7% of total measured *var* gene transcripts in unselected 3D7 parasites, increasing to 80% after seven rounds of PAM1·4 selection. In contrast, PAM8·1 selection did not affect the proportion of *var2csa* transcripts in 3D7 (Figure 1g). The HB3 genome contains two *var2csa* paralogs, *var2csa-A* and *var2csa-B* (13). In unselected HB3 parasites, *var2csa-A* transcripts constituted 1% of the measured *var* transcripts, increasing to 28% and 4% after seven rounds of selection by PAM1·4 and PAM8·1, respectively (Figure 1h). Transcript levels of the *var2csa-B* gene increased from 23% to 57% and 92% after seven rounds of selection by PAM1·4 and PAM8·1, respectively (Figure 1h). No *var* transcript other than *var2csa* showed marked changes, and *var2csa* was therefore the dominant transcript in all the parasites following antibody selection. The dominance of *var2csa-B* transcripts relative to *var2csa-A* in HB3 after PAM8·1 selection, suggested that the DBL3-X domain in the protein encoded by *var2csa-B* might be of the FCR3-type recognized by PAM8·1, whereas the corresponding domain encoded by *var2csa-A* might be of the 3D7-type (PFL0030 c) not recognized by PAM8·1 (2). However, *var2csa-A* and *var2csa-B* encode an identical amino acid sequence in the region spanning the PAM8·1 epitope, and this sequence was of the FCR3-type (Figure 1i). The *var2csa-A* : *var2csa-B* transcripts may therefore reflect a founder effect.

## DISCUSSION

The VSA subset VSA_PAM_ is expressed by *P. falciparum* involved in pregnancy-associated malaria (PAM). VSA_PAM_-specific IgG mediates acquired immunological protection against PAM, and the PfEMP1 variant VAR2CSA appears to be the main or only target of these antibodies. VAR2CSA is therefore the leading candidate for development of vaccines against PAM.

We used monoclonal human IgG antibodies to select erythrocytes infected by two genotypically distinct laboratory *P. falciparum* clones derived from nonpregnant donors for expression of VSA_PAM_. Parasites acquiring expression of VSA_PAM_ following selection showed increased levels of transcripts encoding the PfEMP1 variant VAR2CSA, which appears to be the only PAM-type VSA in the *P. falciparum* genome. The results obtained in the study are important for several reasons.

First they support the hypothesis that all *P. falciparum* parasites have the capacity to express VSA_PAM_. This hypothesis is supported by previously published data that all *P. falciparum* genomes appear to contain at least one paralog of the gene encoding the only known VSA_PAM_-type antigen, VAR2CSA (11,14). Furthermore, this gene is selectively transcribed by placental parasites (and following selection for adhesion to CSA *in vitro*) and VAR2CSA is expressed on the IE surface (15–17).

Second, they indicate that *P. falciparum* parasites regularly and spontaneously switch to expression of VSA_PAM_ in the absence of an external signal, for example pregnancy-associated hormonal changes. It has been speculated that switching to VSA_PAM_ expression requires signals from the pregnant host, for example hormones, and that selection therefore might not be possible unless the parasite is derived from such a host. It has also been argued that selection of parasites by panning on CSA *in vitro* might result in expression of antigens of dubious relevance to the antigens expressed as a result of *in vivo* selection occurring in the pregnant woman. Our data show that switching to expression of genuine VSA_PAM_ can occur *in vitro* in the absence of external signals, in line with other recent evidence (18). By extension, these findings suggest that switching to VSA_PAM_ *in vivo* also occurs spontaneously regardless of the pregnancy status of the host. In a pregnant host, such parasites will often be at a selective advantage (because of the frequent absence of VSA_PAM_-specific immunity in women of low parity) (19), whereas they appear to be unable to survive in a nonpregnant host (20).

Third, our results support the hypothesis that although interclonal variation in VAR2CSA is the result of antibody-driven positive selection, the diversity of functionally important antibody epitopes in the molecule is constrained (21). Thus, some parasites can express VAR2CSA without being vulnerable to recognition by certain VAR2CSA-reactive antibodies, because of variation in defined parts of VAR2CSA. The persistent nonrecognition by PAM8·1 of VAR2CSA-expressing 3D7 is a case in point. At the same time, the PAM8·1 antibody, originally identified by its reactivity with VSA_PAM_-expressing FCR3-IEs (3), was effectively selected for VSA_PAM_ expression in the genetically unrelated HB3 clone. The significance of these findings is underscored by the fact that PAM8·1 efficiently opsonizes IEs for phagocytosis as well as interferes with IE adhesion to CSA (Barfod *et al.* in preparation). Our study also corroborates existing evidence that PAM1·4 recognizes most if not all VSA_PAM_-expressing parasites by recognizing a functionally highly constrained and conformation-dependent discontinuous epitope in VAR2CSA (3). Like PAM8·1, PAM1·4 is an opsonin and capable of interfering with IE adhesion to CSA (Barfod *et al.* in preparation). Therefore, the PAM1·4 epitope is a highly attractive candidate for development of a PAM-specific vaccine, and its characterization is a matter of the highest priority in our current research. Although the theoretical possibility of another, non-PfEMP1 target of PAM1·4 remains, available evidence point to VAR2CSA.

In conclusion, our evidence support current efforts to develop PAM-specific vaccines based on VAR2CSA and highlight the versatility of human monoclonal antibodies generated from clinically immune donors in these investigations.

## SUPPORTING INFORMATION

**Table S1** Real-time PCR primers for HB3 var genes
