# Supplementary material for: Human monoclonal IgG selection of Plasmodium falciparum for the expression of placental malaria-specific variant surface antigens
Source: Parasite Immunol. 2009 Jun;31(6):341–6. doi: 10.1111/j.1365-3024.2009.01097.x (PMC3001033; doi:10.1111/j.1365-3024.2009.01097.x)
Supplement: Supplementary file 1 [file pim0031-0341-SD1.doc]

# Supplementary Table 1. Real-time PCR primers for HB3 *var* genes

| Gene* | Forward | Reverse |
| --- | --- | --- |
| HB3var1 | AAGTGCAAATGTAAAGATCCTGTCCC | TATCATCTGTTGCGCCCTTGCC |
| HB3var10 | GAAAGAAGCAAGATTTCCACATCGC | ACCACCACTGGTAACATTAGTATCTC |
| HB3var11 | GTTTGACAGGAGCAACATTTCATAC | AGGATTATCGTTATCGTAACCACTGG |
| HB3var12 | GGCCAACATACTTACTGGCAAAGAC | GTACTACCACTACCACTTGGTGTGAC |
| HB3var13 | GGTGAATTGGAAGGAAATTTGAAGG | TTCCATTTGCACTTCTGCAC |
| HB3var14 | ACCACACACGCAAGAAACGG | TCACATGCCTTTGTGAGATTTTCC |
| HB3var15/HB3var40 | CTTGACGACTACTACATACAACAG | GGATAATTCTATTCCATTGGCCTTC |
| HB3var16 | CGTACAGAATCGACACCACCAAATCC | ACGTTCTTCTGTTCCATTTCCACAAG |
| HB3var17 | CGAACAATCATTCCAATGCTACTG | TCTTCTCGGAGGCATATAAGC |
| HB3var18/HB3var44 | ACAGAGGTAACAGAGACAACTAC | GTACTGTCACCTATACATTTCCAAC |
| HB3var19 | GACCTAAAGAAACAACACCAGAAG | GTACACTGACCTCCATATTCATC |
| HB3var1csa | GAAAGTGAGAACAGTCCAAGAAATG | TACTGGCGTAGAAGGACCAC |
| HB3var2 | GTTCATCAACTCCTTCGGTTCC | GCCAAATCTCCTTTCAAATGATGTCC |
| HB3var20 | GCGTTGAAAGGAGATTTGAAGAAAG | GTTCTACATCGTTTCCACTTAACTC |
| HB3var21 | CGAACATTCCAATGATAGTCGTC | GCCCTTAGTCTTCCATATAGTTCC |
| HB3var22 | ACGATCAACAAACACCACAG | CTCTTATTACATTCTGCTCCTTCTG |
| HB3var23/HB3var41/HB3var42 | ACGCATGTACCCTCAAGTATGGAAG | TATGGCACCGTCCTTATCACCACC |
| HB3var24 | CAAGATGCTTGTACCCTCAAATATGC | GGCGGAACACAAATACTACCACTATC |
| HB3var25 | AAGACGACGAGGAAGACGACGAAG | CGTGTAAGTATTGCGTCCGCTATTTC |
| HB3var26 | GGTGCTGCCCTAGAACCTAGTAAG | GATGGCATGGATCATTTGGTGTTTC |
| HB3var27 | GTCGTAACAGTGAAAGGAATCCG | GCACATGCTCCACTTTCTTTAAC |
| HB3var28 | GGAACTTTGTCACAAGCAACAAATC | TGCACGGATACCTTTCATTGTTAG |
| HB3var29 | GATGTAAATGTGTGCGAAACAG | ATACACAAACCACCCTTATCAC |
| HB3var2csaA | TGATGGTGTTCCTCAATTTCTTCG | CCTTTCTTAGTATCACAACTCCCAG |
| HB3var2csaB | GATCAATTGAAAGGTTCT | AGTACCTTGTCCTTTTCTCGT |
| HB3var3/HB3var46 | CCACTGTTGATACACCTTCC | CATACTGCTTATTGTCGTCCTTAC |
| HB3var30 | GGCTGAACTATTTAAAGAAACGGAC | CACAAATACTACCACTAGCATCAC |
| HB3var31 | CGTATGGGTAAAGGTTGCACTGACTG | ACCAACAGCACCACTACCACTAGC |
| HB3var32 | GGAGTGGGTGAAATTGATCATACAAG | CTGTAACTTTCCTTTCAACGCCTC |
| HB3var33 | ACCTATTTAAAGACGGCACTAC | CGACCTTTGGCTTCACTATC |
| HB3var34 | GCATCGTTAGAATATCCACCTATTG | CCTTAAGTATTTCCTCCGCTATTTC |
| HB3var35/HB3var51 | ATCTGTAAACCCAACCCTCCCTGC | TGAAAAGGTCCACGGGGGATTG |
| HB3var36 | CTTACCATCGACAATCTCACAAAGG | CACTACCACTTGTGACACACTTCC |
| HB3var37/HB3var48 | CTCCTCCATTGTATAAGGAGGCG | GTATTGTATTCACCATGTTGCAGGC |
| HB3var39/HB3var49 | GGCATTTGAAGGACGCAACATATTC | TCGAACGTCGGGTCTATCTTTAC |
| HB3var4 | AGGAGGAAGATGACGAAGAAGAGGAG | TTTCGCACACATCCACAGTGTCTTG |
| HB3var47 | GCCAACAGATGCAAAAACTG | TTCATCTTCCTGTGCAGGTG |
| HB3var5 | TGACAAAAGCACCACTTTGG | CCTTCTTCTTCCGTTCACGA |
| HB3var50 | AACAACGAAGTCGAGGAGACGGAG | TGAGAGAACACGCTGCTTGGAG |
| HB3var6 | TGATACATCAGACGATGAAGATGAC | CCAATAAGTTTCTTCACCATTTCGC |
| HB3var7 | GCAATTTGGATGAAGCCTGCCAAC | GGGATACACAAACCACCAGTACCAC |
| HB3var8 | GGAGGTCGTATTGAGGATACAACTG | CGTCCTTCAAACTTCCATGCAAATC |
| HB3var9 | GGTGAGGATGGTATTGATAAACGAAG | CCTCTTTATATGTTTGAGCCTCAGCC |
| PFHG_04621.1 | GTACATGCCCAAGTACAAAATGAAG | CCCATTTCCTCACTAGAAAATGTTG |
| PFHG_05483.1 | TCCACAGAAGACACCGAATC | CGGAGGAAGTTACATCAGTTGTATC |

*: Gene nomenclature as in (10) and the HB3 annotation by Broad Institute ([http://www.broad.mit.edu](http://www.broad.mit.edu/))
